# Lysosomal dysfunction and autophagy blockade contribute to autophagy-related cancer suppressing peptide-induced cytotoxic death of cervical cancer cells through the AMPK/mTOR pathway

**DOI:** 10.1186/s13046-020-01701-z

**Published:** 2020-09-22

**Authors:** Yang Yang, Qi Wang, Dongjian Song, Ruirui Zen, Lei Zhang, Yingjun Wang, Heying Yang, Da Zhang, Jia Jia, Jiao Zhang, Jiaxiang Wang

**Affiliations:** 1 Department of Pediatric Surgery, The First Affiliated Hospital of Zhengzhou University, Zhengzhou, 450052 Henan China; 2 Departemnt of Oncology, The First Affiliated Hospital of Zhengzhou University, Zhengzhou, 450052 Henan China

**Keywords:** Cervical cancer, ARCSP, Autophagic flux, Nonfused autophagosome, Autophagy-related cytotoxic death

## Abstract

**Background:**

Autophagy is an intracellular process through which intracellular components are recycled in response to nutrient or growth factor deficiency to maintain homeostasis. We identified the peptide autophagy-related cancer-suppressing peptide (ARCSP), a potential antitumor peptide that disrupts intracellular homeostasis by blocking autophagic flux and causes cytotoxic death.

**Methods:**

The proliferative ability of ARCSP-treated cervical cancer cells was examined by the CCK8, EdU, and colony formation assays. The TUNEL assay was used to detect apoptosis. Mitochondrial function was evaluated based on the mitochondrial membrane potential. Autophagic flux was detected by immunofluorescence and confocal microscopy. The autophagy-related proteins AMPK, Raptor, mTOR, p62, LC3B, atg7, Rab7, LAMP1, LAMP2, and cathepsin D were detected by Immunoblotting. The antitumor effect of ARCSP was explored in vivo by establishing a transplant tumor model in nude mice.

**Results:**

The results demonstrated that ARCSP induced cell death and inhibited proliferation. ARCSP induced AMPK/mTOR activation, resulting in the accumulation of the proteins LC3B, p62 and Atg7. ARCSP also blocked autophagosome-lysosome fusion by inhibiting endosomal maturation and increasing the lysosomal pH. The accumulation of nonfused autophagosomes exacerbated cytotoxic death, whereas knocking down Atg7 reversed the cytotoxic death induced by ARCSP. ARCSP-treated cells exhibited increased cytotoxic death after cotreatment with an autophagy inhibitor (Chloroquine CQ). Furthermore, the tumors of ARCSP-treated nude mice were significantly smaller than those of untreated mice.

**Conclusions:**

Our findings demonstrate that ARCSP, a novel lethal nonfused autophagosome inducer, might cause mitochondrial dysfunction and autophagy-related cytotoxic death and is thus a prospective agent for cancer therapy.

## Background

Cervical cancer is the fourth most common malignancy in women worldwide and the third most common cause of cancer death in low-income and middle-income countries [1]. The combination of cisplatin and paclitaxel has been the standard of treatment for advanced cervical cancer for many years, but its efficiency is only 20 to 30% [2]. Tumor cells have the ability to evade apoptosis, which can cause tumors to become resistant to chemotherapeutic drugs [3]. Therefore, regulation of cell death may be an alternative therapeutic strategy for cervical cancer.

Our previous study identified a peptide (detected at m/z 6455.5 Da by proteomics) that is upregulated in healthy children and downregulated in Wilms tumors [4]. This peptide consists of 55 amino acids and is the smallest member of the apolipoprotein C family. Two forms of apolipoprotein C1 (APOC1) have been found in the plasma, a mature peptide at m/z 6630 Da (57 amino acids) [5] and a shear peptide at m/z 6432 Da (55 amino acids) [6]. These two forms of peptides play an important role in the body, participating in different physiological and pathological reactions. The levels of the peptide at m/z 6432 Da are significantly reduced in the serum of non-small cell lung cancer [7], papillary thyroid carcinoma [8], triple negative breast cancer [9], and nephroblastoma [4, 10] patients compared to normal controls, indicating that it may be a potential biomarker for tumors. These specific protein markers play an important role in the diagnosis and treatment of malignant tumors [11]. For example, the peptide at m/z 6432 Da can inhibit the proliferation of nephroblastoma cells by regulating the Wnt/β-catenin pathway and has a potential antitumor effect [4]. Moreover, we found for the first time that the peptide at m/z 6432 Da can inhibit the proliferation of cervical cancer cells by regulating autophagy; thus, we temporarily named the peptide at m/z 6432 Da autophagy-related tumor suppressor peptide (ARCSP). In this study, we further explored the relationship between ARCSP-induced cell death and autophagy.

Autophagy is a lysosomal-mediated form of degradation in cells. The process of autophagy includes the formation of autophagosomes, autophagosome-lysosomal fusion, and autophagolysosomal degradation [12]. During degradation, an acidic pH and the enzymatic action of lysosomal hydrolase leads to destruction of the inner membrane of autophagosomes and the elimination of autophagolysosomes, thereby maintaining cell homeostasis [13]. Abnormalities in the clearance process are associated with a variety of pathologies, including oncogenesis, neurodegenerative diseases, metabolic diseases, and immune-related diseases [14]. It has been found that abnormal accumulation of autophagosomes and impaired autophagolysosomes can induce tumor cell death [15–17]. Sustained and excessive induction of autophagy by anticancer drugs induces irreversible destruction of cellular contents, ultimately leading to cell death [18]. Because ARCSP is a cytotoxic peptide, it, like other peptides, may cause cellular stress [19]. We hypothesized that ARCSP is an antitumor peptide and confirmed our conjecture by testing its antiproliferative effect on cervical cancer, esophageal cancer, neuroblastoma, and kidney cancer cells. It is reasonable to assume that autophagy may be involved in ARCSP-induced cytotoxic death.

In this study, we found that ARCSP induced the accumulation of nonfused autophagosomes by activating the AMPK/mTOR pathway, inhibiting proliferation and causing cytotoxic death of cervical cancer cells. ARCSP-induced cytotoxic death of cells may have mainly resulted from increased ROS levels caused by mitochondrial dysfunction and accumulation of a large number of nonfused autophagosomes. ARCSP had a cytotoxic effect on cervical cancer cells both in vitro and in vivo. These findings indicate that ARCSP is a promising antitumor peptide.

## Materials and methods

### Cell culture

Human cervical adenocarcinoma cells (HeLa cells), human cervical adenocarcinoma intestinal metastasis cells (CaSki cells), human normal cervical epithelial cells (HCerEpiC cells), and human normal liver epithelial cells (LO-2 cells) were purchased from the Cell Bank of the Shanghai Institute of Chinese Academy of Sciences. These cell lines were maintained in RPMI 1640 with 10% FBS and incubated in a humidified incubator (5% CO_2_ at 37 °C); the medium was changed every 2 days.

### Synthesis of ARCSP

**Fig. 1:**
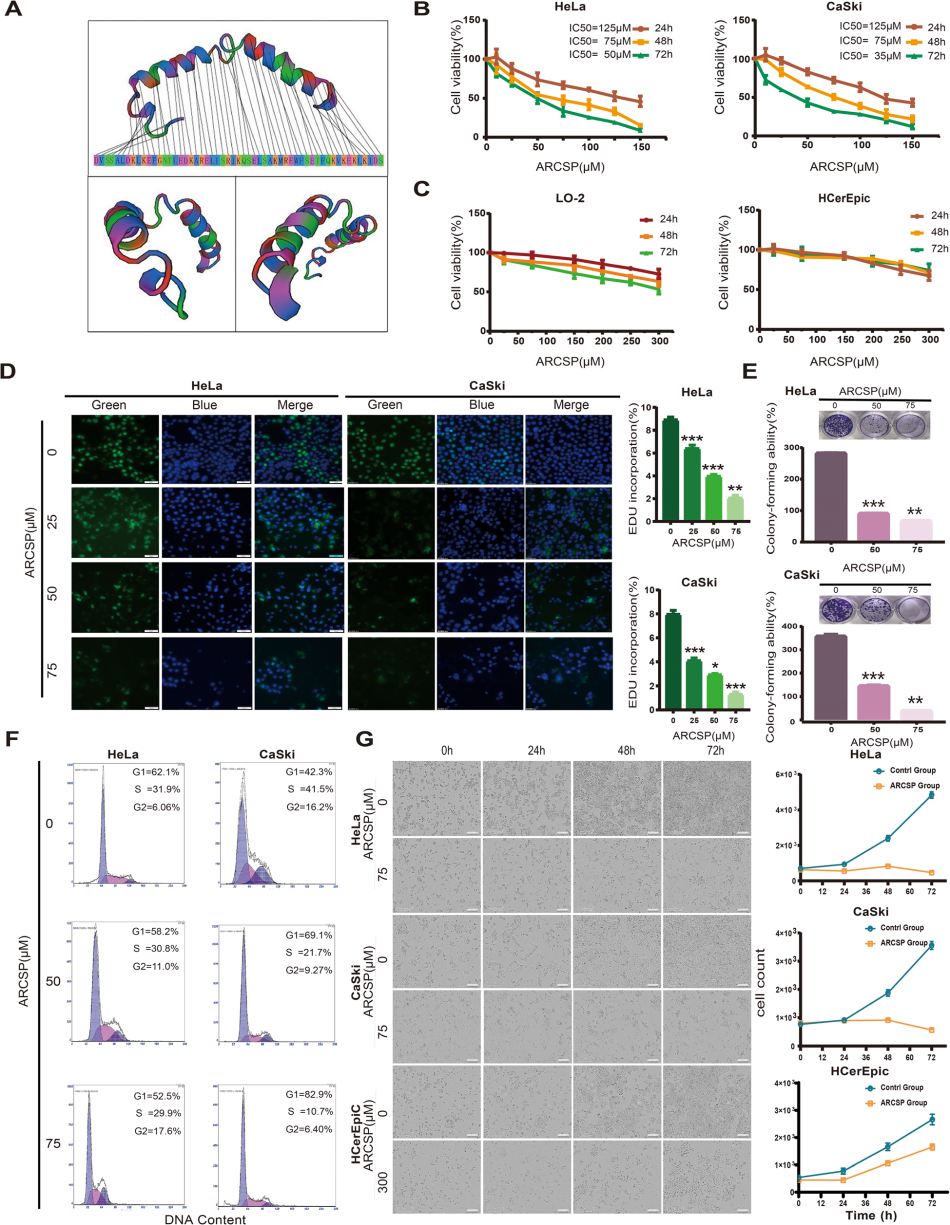
ARCSP inhibits the proliferation of cervical cancer cells and has low cytotoxicity in normal cells. **a** Model of the secondary structure of ARCSP. **b** Cervical cancer cell lines were treated with ARCSP (0–150 μM) for 24–72 h, and cell viability was measured by the CCK8 assay. **c** Normal cell lines (LO-2 and HCerEpic cells) were treated with ARCSP (0–300 μM) for 24–72 h, and cell viability was measured by the CCK8 assay. **d** Cells were treated with ARCSP (0–75 μM) for 48 h, and the proliferation of cells was measured by the EdU proliferation assay. Scale bar = 50 μm. The histograms show the quantified EdU incorporation data, which were calculated using ImageJ software. **e** Cells were treated with ARCSP (0–75 μM) for 14 days, and clone-forming ability was determined by the cell colony formation assay. The histograms show the quantified results of the colony formation assay, which were calculated using ImageJ software. **f** After 48 h of treatment with ARCSP (0–75 μM), cell cycle distribution was analyzed by FACS; the ratios of cells in G1, S, and G2/M phases are shown on the right. **g** After 72 h of continuous treatment with ARCSP (75 μM, 300 μM), the number of cells was significantly reduced. Scale bar = 300 μm. The data are expressed as the mean ± SD; ^*^P < 0.05, ^**^P < 0.01, ^***^P < 0.001. ns, not significant

ARCSP was synthesized by solid-phase synthesis of FOMC-protected amino acids and purchased from Life Protein Biotechnology Company (Beijing, China). Its sequence was DVSSALDKLKEFGNTLEDKARELISRIKQSELSAKMREWFSETFQKVKEKLKIDS (Table 1), and the secondary structure of ARCSP was predicted and modeled by the molecular modeling server (SWISS-MODEL, Switzerland) (Fig. 1a). The purity of ARCSP was more than 95%, as was confirmed by high-performance liquid chromatography (HPLC) analysis (Fig. S1A), and mass spectrometry (MS) was performed to characterize the peptide (Fig. S1B). In addition, a part of ARCSP was labeled with fluorescein isothiocyanate (ARCSP-FITC) for Immunofluorescence. The mother liquor (2 mg/mL or 300 μM) was prepared in RPMI 1640 with 10% FBS and then diluted to the working concentration with different media.

**Table 1:** The amino acid sequence and related information of ARCSP

Sequence interpretation and physiochemical properties of ARCSP	
Single letter code	DVSSALDKLKEFGNTLEDKARELISRIKQSELSAKMREWFSETFQKVKEKLKIDS
Triple letter code	Asp -Val -Ser -Ser -Ala -Leu -Asp -Lys -Leu -Lys -Glu -Phe -Gly -Asn -Thr -Leu -Glu -Asp -Lys -Ala -Arg -Glu -Leu -Ile -Ser -Arg -Ile -Lys -Gln -Ser -Glu -Leu -Ser -Ala -Lys -Met -Arg -Glu -Trp -Phe -Ser -Glu -Thr -Phe -Gln -Lys -Val -Lys -Glu -Lys -Leu -Lys -Ile -Asp –Ser
Number of residues	55
Molecular weight	6432.41 g/mol
Extinction coefficient	5690 M^−1^ cm^−1^
Iso-electric point	pH = 9.4
Net charge at pH 7	1
Estimated solubility	Good water solubility

### Reagents and antibodies

The reagents used in this study were purchased from Chinese suppliers: Z-VAD-fmk (CSNpharm, CSN15936, Chicago, USA), CQ (Sigma-Aldrich, C6628, St. Louis, MO, USA), NAC N-acetyl-L-cysteine (Beyotime, S0077, Shanghai, China), AO (Sigma-Aldrich, A6014), 3-MA (Selleck, S2767, Houston, Texas, USA), rapamycin (Selleck, S1039), Cisplatin (Selleck, S1166), and Dorsomorphin (Selleck, S7846). The following antibodies were also purchased from Chinese suppliers: GAPDH (Proteintech, 10,494–1-AP, Wuhan, China), cathepsin D (Proteintech, 21,327–1-AP), PCNA (Proteintech, 10,205–2-AP), Ki-67 (Proteintech, 27,309–1-AP), LAMP2 (Proteintech, 66,301–1-lg), p70s6K (Proteintech, 14,485–1-AP), tubulin (Proteintech,1124–1-AP), P21(Proteintech, 10,355–1- AP), cleaved-caspase9 (Asp175) (CST, 52873, Beverly, MA, USA), cleaved caspase 8 (Asp391) (CST,9496), Bax (CST 2774), Bid (CST,2003), Raptor (CST,2282), p-raptor(Ser792) (CST,2083), Rab7 (CST,9367), AMPK (CST, 5832), p-AMPK (CST, 2535), mTOR (CST, 2983), p-mTOR (CST,5536), p-p70s6K (CST,9234), LC3B (CST, 3868), LAMP1 (CST 15665), Atg7 (CST,8558), p62 (CST, 88588).

### Cell viability assay

Cells were cultured in 96-well plates with ARCSP for different durations. Then, the cells were washed, 110 μL of fresh medium containing CCK8 reagent (Dojindo, CK 04, Tokyo, Japan) was added, and the cells were incubated for 1–2 h. The absorbance was measured at 450 nm by a full-wavelength microplate reader (Molecular Devices, SpectraMax M5, Silicon Valley, USA). The effect of ARCSP on the survival rate of cervical cancer cells was evaluated.

### Lactate dehydrogenase (LDH) release assay for the evaluation of cytotoxicity

The cells were incubated with ARCSP in 96-well plates for 48 h, and LDH activity was detected according to the instructions of the LDH release kit (Dojindo, CK 12). The absorbance was measured at 490 nm by a full-wavelength multifunction microplate reader. Cervical cancer cell injury was assessed after ARCSP intervention based on LDH release.

### 5-Ethynyl-2′-deoxyuridine glycoside (EdU) proliferation assay

After incorporation of EDU, cell proliferation was measured using the BeyoClickTM EdU-488 Cell Proliferation Assay Kit (Beyotime, C0071S). The cells were fixed, permeabilized, and labeled with EdU according to the manufacturer’s instructions after ARCSP treatment for 48 h. The nuclei were stained with Hoechst 33342 (1 μg/ml) for 10 min at room temperature. The proportion of cells showing EdU incorporation was measured under an inverted fluorescence microscope (Olympus, IX73, Tokyo, Japan).

### Cell colony formation assay

Briefly, 1000 cells were cultured in 24-well plates and treated with ARCSP. After 14 days, the cells were fixed with 4% paraformaldehyde for 15 min, stained with crystal violet for 15 min, and washed three times. The number of colonies with a cell size > 1 mm was counted.

### Cell apoptosis

Cells were cultured, treated for 48 h, collected, washed with PBS, resuspended and incubated in FITC Annexin-V/PI solution (Biolegend, 640,914, USA) for apoptosis analysis. At least 10,000 live cells were analyzed on a flow cytometer (Beckman, Miami, USA).

### TUNEL assay

The cells were cultured in 96-well plates, incubated with ARCSP for 48 h, fixed in 4% paraformaldehyde and then permeabilized in 0.2% Triton X-100. TUNEL staining (Roche, 11,684,817,910, USA) was performed according to the manufacturer’s instructions and observed under a fluorescence microscope (Leica, DMi8, Wetzlar, Germany).

### Cell cycle

The cell cycle was evaluated by using PI (Becton Dickinson, 550,825, New Jersey, USA). Briefly, 2 × 10^5^ cells were cultured in 6-well plates and incubated overnight (the serum was removed to synchronize the cells). After ARCSP treatment for 48 h, the cells were collected, fixed in 70% ethanol overnight at 4 °C, and then stained with 0.5 ml of PI/RNase staining solution for 15 min. DNA content was analyzed by flow cytometry to determine ratio of cells at each stage of the cell cycle. At least 20,000 viable cells were analyzed on a flow cytometer (Beckman).

### Measurement of ROS levels

Cells were treated with ARCSP for 48 h, the oxidation-sensitive probe dichlorodihydrofluorescein diacetate (DCFH-DA, Beyotime, S0033) was added, and the cells were incubated for 30 min and then imaged under an inverted fluorescence microscope. To quantify the intensity of the probe, flow cytometry (Becton Dickinson) was used to detect changes in intracellular ROS levels.

### Measurement of the mitochondrial membrane potential

Cells were cultured in 96-well plates, incubated with ARCSP-FITC for 48 h, and then washed with PBS. Then, 100 μL MitoTracker Red CMXRos (60 nM) was added for 30 min in a humidified incubator. The nuclei were stained with Hoechst 33342 (1 μg/ml) for 10 min at room temperature and observed under a fluorescence microscope.

### Intracellular ATP assay

Cells were uniformly seeded in 96-well plates and treated with ARCSP for 48 h. Then, the ATP level in the cells was detected according to the instructions of the Luminescent ATP detection assay kit (Abcam, Cambridge, MA, USA).

### Transmission electron microscopy

Autophagosomes were observed by transmission electron microscopy (TEM). Cells were incubated with ARCSP for 48 h, collected, fixed in 2.5% glutaraldehyde (pH 7.3–7.4) at 4 °C overnight and treated with 1% osmium tetroxide for 2 h. Then, the samples were dehydrated in ethanol (70, 80, 90 and 95%) and propylene oxide, embedded, cut into 50 nm-sections, and stained with 3% uranyl acetate and lead citrate. Images were taken under a transmission electron microscope (TOSHIBA, Tokyo, Japan).

### mRFP-GFP-LC3 puncta assay

Cells were plated in a 24-well plate, transfected with mRFP-GFP-LC3 adenovirus (HanBio Technology, Shanghai, China) for 4 h, incubated with ARCSP for 48 h and observed under a confocal microscope (Zeiss, LSM880). mRFP was used to label and track LC3. Attenuation of GFP expression indicated that lysosomes had fused with autophagosomes to form autophagolysosomes, and GFP was quenched due a change in the pH, at which point only red fluorescence was detected. After microscopic imaging, the red and green fluorescence images were merged; the yellow puncta were considered autophagosomes (RFP^+^GFP^+^), and the red puncta were considered autophagolysosomes (RFP^+^GFP^−^). The intensity of autophagy flux was assessed by counting the number of yellow and red puncta. ARCSP treatment increased the numbers of yellow and red puncta, indicating that it increased autophagic flux; when the number of yellow puncta but not red puncta increased in the cells or when the numbers of yellow and red puncta decreased in the cells, autophagic flux was blocked.

### Lyso-Tracker Red staining

Cells were cultured in 96-well plates, incubated with ARCSP-FITC for 48 h, and then washed with PBS. Then, 100 μL LysoTracker Red (60 nM) was added, and the cells were incubated for 40 min in a humidified incubator. The nuclei were stained with Hoechst 33342 (1 μg/ml) for 10 min at room temperature, and the cells were observed under a fluorescence microscope.

### AO staining

Cells were treated with ARCSP for 48 h, incubated with acridine orange (AO) (1 μg/ml) for 15 min. The nuclei were stained with Hoechst 33342 (1 μg/ml) for 10 min at room temperature, and the cells were observed under a fluorescence microscope.

### Assessment of autophagy by immunostaining

After 48 h of stimulation with ARCSP, cells were fixed with 4% paraformaldehyde for 20 min and permeabilized with 0.1% Triton-PBS for 15 min. The cells were incubated with LC3B and p62 antibodies overnight at 4 °C on a horizontal shaker. Then, the cells were incubated with an Alexa Fluor® 488-conjugated goat anti-rabbit IgG secondary antibody and an Alexa Fluor® 594-conjugated goat anti-mouse IgG secondary antibody for 1.5 h at room temperature, washed with PBS and stained with DAPI (10 μg/ml) for 10 min. Photographs were taken under an inverted fluorescence microscope. Cell-induced autophagy was evaluated by observing LC3B and p62 staining, and the images were quantitatively assessed with ImageJ software.

### RNA extraction and quantitative real-time polymerase chain reaction (PCR)

p62 RNA was extracted from HeLa and CaSki cells by using RNAiso Plus (Takara, 9109, Tokyo, Japan), and 1 μg of total RNA was reverse transcribed using a PrimeScript RT kit (Takara, RR047A) to detect relative mRNA levels. Quantitative real-time PCR (Takara, RR820A) was performed on the Quantitative PCR System (Applied Biosystems 7500, California, USA). The Ct values obtained from different samples were compared using the 2^-ΔΔCt^ method. GAPDH served as an internal reference gene. The sequences of primer pairs were as follows: human p62, forward primer: CCGTCTACAGGTGAACTCCAGTCC; reverse primer: AGCCAGCCGCCTTCATCAGAG; human GAPDH, forward primer: CCCACTCCTCCACCTTTGAC; reverse primer: TCTTCCTCTTGTGCTCTTGC (Sangon Biotech).

### Short hairpin RNA (shRNA) transfection

The shRNA sequence was as follows: human Atg7 (sh-Atg7): TTCTCCGAACGTGTCACGTAA (HanBio Technology). The cells were transfected with lentivirus expressing sh-Atg7 or sh-nc, and the knockdown efficiency of the target protein Atg7 was determined by Western blotting. Then, after treatment with ARCSP for 48 h, the proliferation of the cells was detected by the CCK8 assay, colony formation assay and Western blotting.

### Western blotting

Cells were lysed in RIPA buffer (Beyotime, P0013). The protein concentration was quantified by using the BCA Protein Assay Kit (Thermo Fisher Scientific Pierce, 23,225, California, USA). The proteins were electrophoresed on 8–15% SDS polyacrylamide gels and then transferred to PVDF membranes (Millipore, ISEQ00010 Burlington, USA). The membranes were placed in 5% skim milk for 2 h at room temperature and incubated with antibodies overnight at 4 °C. Finally, the membranes were incubated with a horseradish peroxidase (HRP)-conjugated antibody for 2 h at room temperature. Target proteins were detected by using an enhanced ECL kit (Millipore, WBKLS0100). GAPDH antibody was used as a control for whole-cell lysates.

### In vivo antitumour effect

BALB/c immunodeficient nude mice (Nu/Nu) mice (6–8 weeks old) weighing 20–22 g were purchased from Beijing Weitong Lihua Experimental Animal Technology Co. Ltd. (China). The animal protocol performed in this study was approved by the Animal Care and Use Committee of the First Affiliated Hospital of Zhengzhou University. HeLa cells (1 × 10^6^ cells) were injected into the right axillae of the nude mice (five mice per group). When the tumor volume was 50 mm^3^, the mice were randomly divided into two groups: the control group and the ARCSP (5 mg/kg) treatment group. ARCSP was dissolved in 0.9% physiological saline. The control group was given the same concentration of physiological saline. ARCSP was administered continuously for 18 days by intraperitoneal injection. The tumor size and body weight of each mouse were measured every 3 days during ARCSP treatment. At the end of the experiment, the mice were sacrificed after anesthesia. The tumors were immediately fixed in 10% formalin and then embedded in paraffin. Tumor sections were selected for immunohistochemical staining of proteins including Ki67, PCNA, LC3B, and Bax. Images were taken under a microscope (Olympus BX51).

### Statistical analysis

Data were obtained from at least three independent experiments and are presented as the mean ± SD. Statistical analysis was performed using GraphPad Prism software 7.0 (GraphPad Software, La Jolla, CA, USA). The nonparametric Mann-Whitney test (for data that were not normally distributed) or Student’s t-test (for data that were normally distributed) was used to measure the differences between the two groups. One-way analysis of variance (ANOVA) was used for comparisons between multiple sets of quantitative data. Pairwise comparisons were performed using the LSD-t test. *P* < 0.05 was considered statistically significant.

## Results

### ARCSP inhibits the proliferation of cervical cancer cells and has low cytotoxicity in normal cells

To study the effects of ARCSP on the growth of human cervical cancer cells, we used HCerEpiC, LO-2, HeLa and CaSki cells. The CCK8, EdU and cell colony formation assays were used to observe the effects of ARCSP on cell proliferation. Our results indicated that ARCSP significantly inhibited the growth of HeLa cells (IC_50_ = 50–125 μM) and CaSki cells (IC_50_ = 35–125 μM) after 24–72 h of treatment (Fig. 1b) and that the growth of cervical cancer cells was reduced in a dose-dependent manner. Moreover, ARCSP did not cause significant toxicity to normal epithelial cells, at a dose (IC_50_ = 300 μM) that was 4 times more than that used in the experiments (IC_50_ = 75 μM; Fig. 1c). The number of living cells that showed by EdU incorporation was significantly reduced by ARCSP in a dose-dependent manner (Fig. 1d). In addition, the cell colony formation experiments showed that the colony-forming ability of the cells was significantly inhibited after ARCSP treatment (Fig. 1e). Cell proliferation was arrested at G2/M phase in HeLa cells and at G0/G1 phase in CaSki cells (Fig. 1f). In addition, quantitative live-cell imaging (Essen, IncuCyteZOOM, USA) showed a time-dependent decrease in the number of cells after treatment with 75 μM ARCSP (Fig. 1g). These results indicated that ARCSP has an inhibitory effect on cervical cancer cells. ARCSP has an IC_50_ = 75 μM in HeLa and CaSki cells. Therefore, the concentration of ARCSP used in the subsequent experiments was 75 μM.

### ARCSP induces apoptosis of cervical cancer cells

**Fig. 2:**
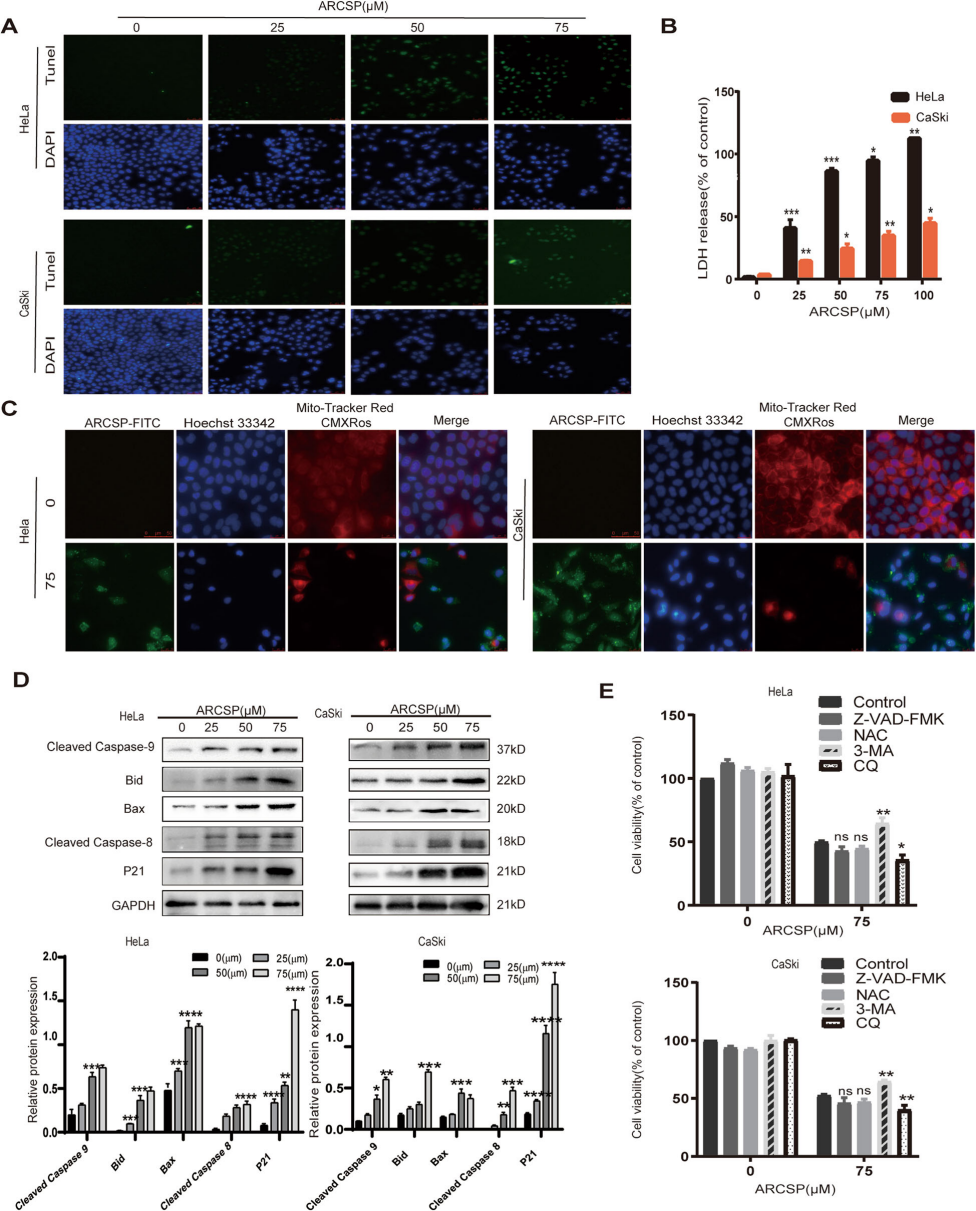
ARCSP induces apoptosis of cervical cancer cells. **a** Cells were treated with ARCSP (0–75 μM) for 48 h, and the apoptosis index was determined by the TUNEL assay. Scale bar = 50 μm. **b** Cells were treated with ARCSP (0–100 μM) for 48 h, and cell membrane damage was detected by the LDH release assay. **c** Cells were treated with ARCSP (75 μM) for 48 h, and the mitochondrial membrane potential was determined by MitoTracker Red staining, Hoechst 33342 (blue) was used to stain the nuclei. Scale bar = 25 μm. **d** Cells were treated with ARCSP (0–75 μM) for 48 h, and the expression levels of cleaved-caspase 9, Bid, Bax, cleaved-caspase 8, and P21 were detected. **e** Cells were cotreated with ARCSP and Z-VAD-FMK (5 μM), NAC (5 μM), 3-MA (20 μM) or CQ (20 μM) for 48 h, and cell viability was determined by the CCK8 assay. The data are expressed as the mean ± SD; ^*^P < 0.05, ^**^P < 0.01, ^***^P < 0.001. ns, not significant

Next, we investigated how ARCSP-induced cervical cancer cell death is regulated. First, HeLa and CaSki cells were treated with different concentrations of ARCSP for 48 h. The TUNEL assay and flow cytometry results showed that ARCSP induced apoptosis (Fig. 2a, Fig. S2A). Furthermore, the LDH release assay also revealed that ARCSP damaged the integrity of the plasma membrane in HeLa and CaSki cells (Fig. 2b). In addition, the mitochondrial membrane potential assay showed that ARCSP exerted a prominent apoptotic effect on cervical cancer cells and induced mitochondrial dysfunction (Fig. 2c). Furthermore, cleaved-caspase8, cleaved-caspase9, Bax, Bid, and P21 accumulated upon ARCSP treatment (Fig. 2d). We also investigated ARCSP-induced production of ROS in HeLa and CaSki cells. ARCSP increased the levels of ROS in cells, as determined by flow cytometry and fluorescence labeling (Fig. S2B, C). NAC is an ROS scavenger that significantly reduces ROS production in cells, but we observed that cotreatment with NAC and ARCSP did not reverse the inhibition of cell proliferation (Fig. S2D). The results indicated that ARCSP damage mitochondria, leading to ROS accumulation, which may have been a cause of cell death.

We also cotreated HeLa and CaSki cells with ARCSP and a series of inhibitors for 48 h. Z-VAD-FMK is a classical caspase inhibitor, but the inhibition of cervical cancer cell proliferation was not reversed by cotreatment with Z-VAD-FMK (Fig. 2e). Interestingly, cotreatment with ARCSP and CQ induced more cell death than treatment with ARCSP alone, indicating that autophagy might also be involved in ARCSP-induced cell death.

### ARCSP induces autophagy, and the AMPK/mTOR signaling pathway may be involved in ARCSP-induced autophagy

**Fig. 3:**
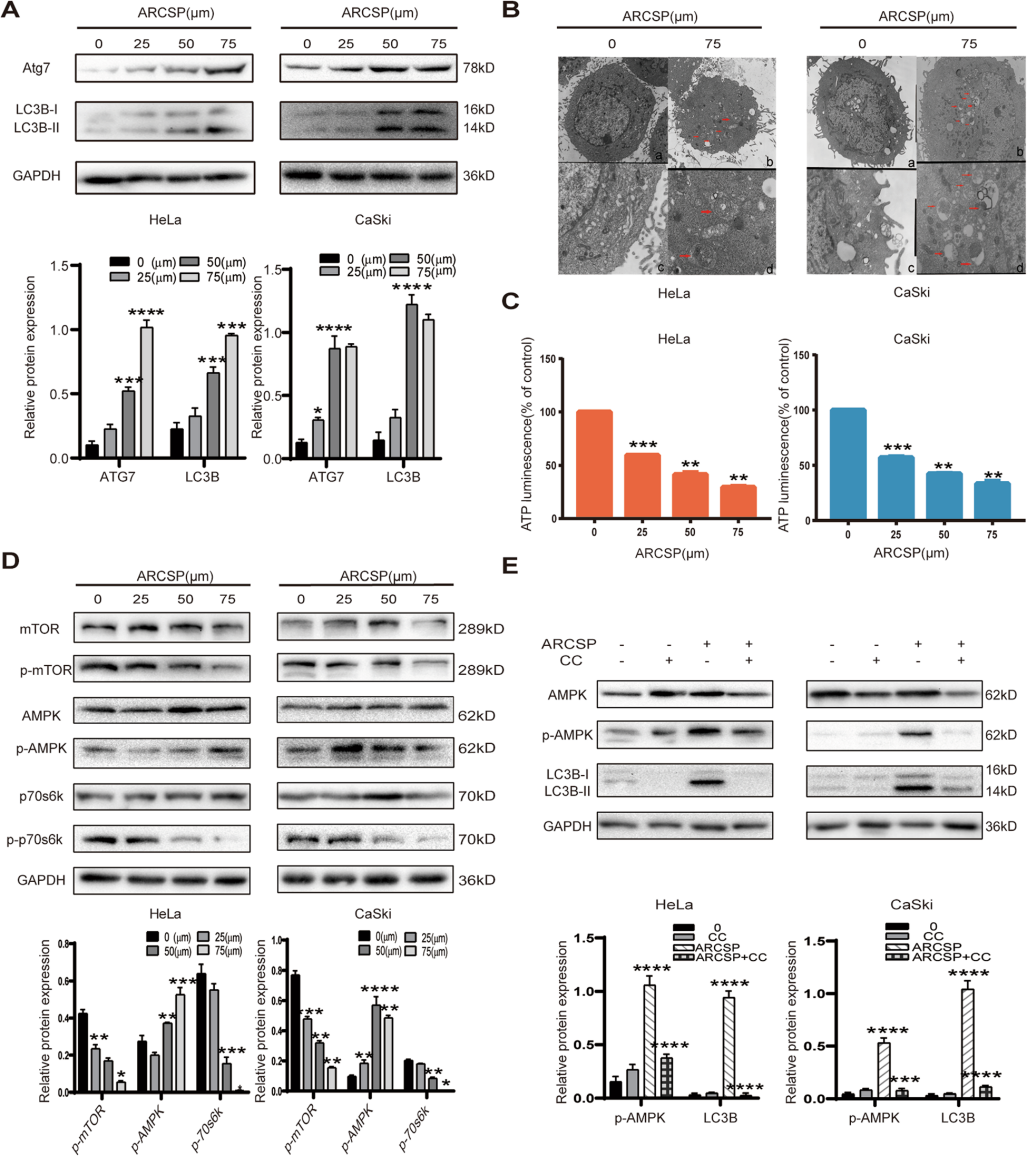
ARCSP induces autophagy, and the AMPK/mTOR signaling pathway may be involved in ARCSP-induced autophagy. **a** After HeLa and CaSki cells were treated with ARCSP (0–75 μM) for 48 h, we detected the expression of LC3B-I, LC3B-II, and Atg7 by Western blotting. **b** Cells were treated with or without ARCSP (75 μM) for 48 h. Autophagosomes were observed by transmission electron microscopy (**a** and **b** 5000×; **c** and **d** 15,000×). Scale bar = 0.1 μm. **c** Cells were treated with ARCSP (0–75 μM) for 48 h, and the ATP assay kit was used to detect intracellular ATP levels. **d** After cells were treated with ARCSP (0–75 μM) for 48 h, the levels of AMPK, p-AMPK, mTOR, p-mTOR, p70s6k, and p-p70s6k were detected by Western blotting. **e** The AMPK inhibitor CC (10 μM) was administered with ARCSP (75 μM) for 48 h, and the expression of LC3B-I, LC3B-II, AMPK, and p-AMPK was detected by Western blotting. The data are expressed as the mean ± SD; ^*^P < 0.05, ^**^P < 0.01, ^***^P < 0.001. ns, not significant

Next, we investigated whether ARCSP induces autophagy in HeLa and CaSki cells. ARCSP treatment induced the transformation of LC3B-I to LC3B-II and upregulated the expression of LC3B-II and Atg7 (Fig. 3a). Fortunately, we were able to observe the bilayer membrane of autophagosomes using TEM (Fig. 3b). More autophagosome aggregation was observed in the ARCSP-treated group than in the untreated control group. These results demonstrate that ARCSP can induce autophagy in cells.

As a key protein of cell growth, mTOR plays an important role in cell metabolism and proliferation. The AMPK/mTOR signaling pathway is the main autophagy-initiating pathway. ARCSP can damage mitochondria and decrease the mitochondrial transmembrane potential (Fig. 2c), leading to decreased production of ATP. We measured ATP levels in cells treated with ARCSP. As expected, compared to untreated control cells, ARCSP-treated cells showed a dose-dependent decrease in ATP production (Fig. 3c). Therefore, we speculate that AMPK might play a critical role in ARCSP-induced autophagy.

Immunoblotting showed that ARCSP positively regulated AMPK and its downstream protein Raptor (Fig. S2E), thereby inhibiting the activity of mTOR. Furthermore, the expression of p70s6k, another downstream protein that plays a key role in regulating autophagy, was significantly inhibited in HeLa and CaSki cells (Fig. 3d). Dorsomorphin (CC), an inhibitor of AMPK, significantly inhibited the expression of the proteins LC3B-II and AMPK in ARCSP-treated cells (Fig. 3e). These data suggest that ARCSP induces autophagy by activating the AMPK/mTOR pathway in HeLa and CaSki cells.

### ARCSP blocks autophagic flux

**Fig. 4:**
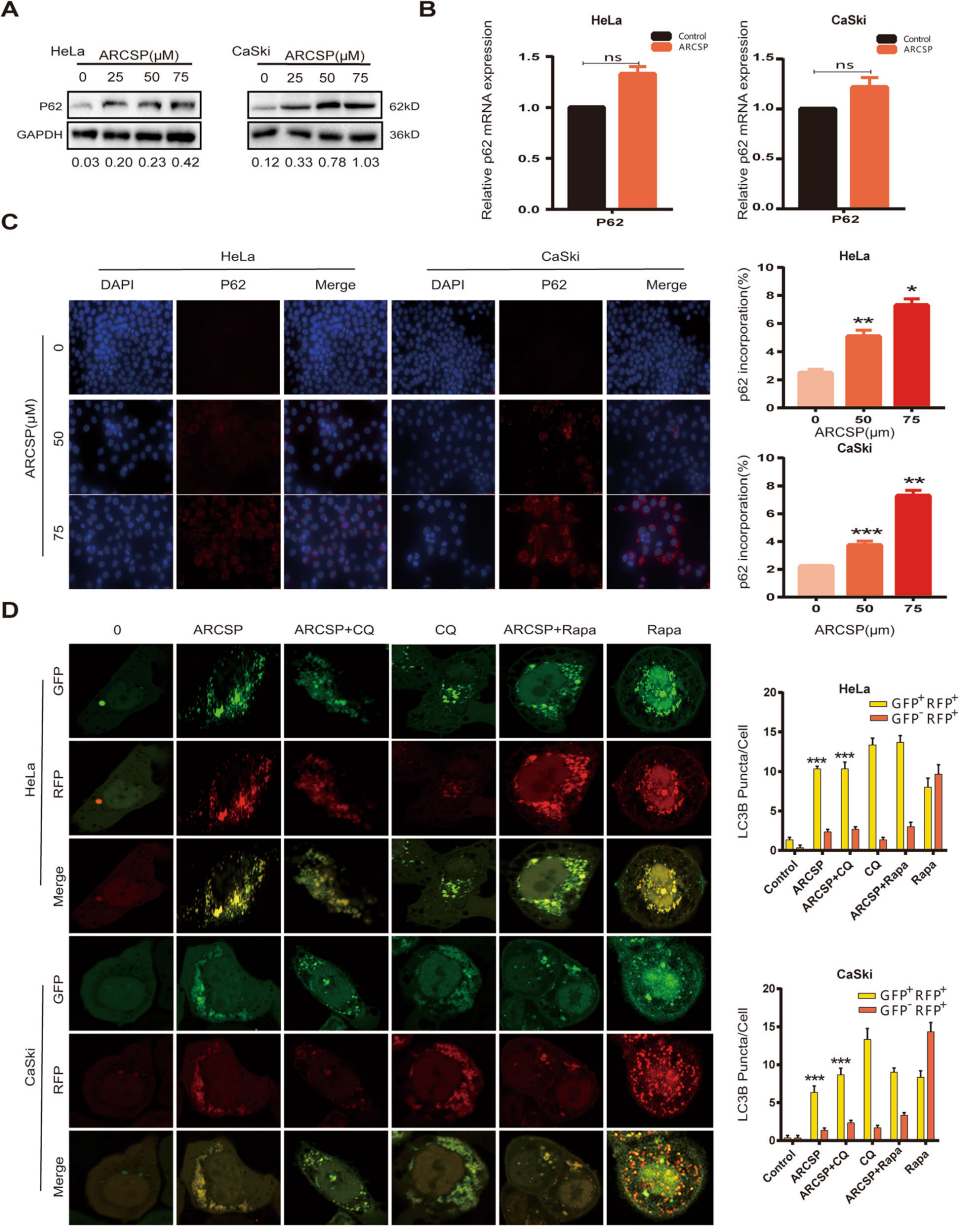
Autophagy flux is blocked by ARCSP. **a** Cells were treated with ARCSP (0–75 μM) for 48 h, and the level of p62 was detected by Western blotting. **b** p62 mRNA expression after treatment with ARCSP (75 μM). **c** Cells were treated with ARCSP (0–75 μM) for 48 h, immunolabeling with p62 (594 red) antibodies. DAPI (blue) was used to stain the nuclei, and the cells were photographed under a fluorescence microscope. Scale bar = 25 μm. The histograms showed quantification results of p62 localization which were calculated using ImageJ software. **d** Cells were transfected with the mRFP-EGFP-LC3 adenovirus, cocultured with ARCSP (75 μM) and Rapa (100 nm) or CQ (20 μM) for 48 h, and analyzed by confocal microscopy. Scale bar = 10 μm. The histograms show the quantification of yellow and red puncta, which was performed using ImageJ software. The data are expressed as the mean ± SD; ^*^P < 0.05, ^**^P < 0.01, ^***^P < 0.001. ns, not significant

The accumulation of autophagosomes (increased levels of LC3B-II) by ARCSP treatment may represent either an increase in the number of autophagosomes or blockade of autophagolysosome formation through the fusion of autophagosomes and lysosomes. Next, we evaluated autophagic flux to clarify whether ARCSP affects the entire process of autophagy. It is well known that the p62 protein is an autophagic substrate, and a reduction in the level of p62 is considered a marker of increased autophagic flux. ARCSP treatment increased the expression of p62 in a dose-dependent manner in HeLa and CaSki cells (Fig. 4a). In addition, there was no significant change in the mRNA level of p62 after 48 h of treatment with ARCSP, indicating that the increase in p62 occurred at the protein level (Fig. 4b). In addition, ARCSP treatment resulted in an accumulation of p62 points (Fig. 4c). Moreover, we investigated the colocalization of endogenous P62 puncta and LC3B (an autophagosome marker) puncta in ARCSP-treated cells. ARCSP treatment resulted in the accumulation of p62 puncta and increased colocalization of these puncta with LC3B puncta (Fig. S3). These results indicate that ARCSP can induce autophagosome accumulation but may block autophagic flux.

We transfected HeLa and CaSki cells with the mRFP-EGFP-LC3 adenovirus to monitor the synthesis of autophagosomes and autophagosome-lysosomal fusion. Autophagosomes were indicated by yellow puncta, and autophagolysosomes were indicated by red puncta (GFP fluorescent protein is sensitive to acidity). When autophagosomes fused with lysosomes, GFP fluorescence was quenched, and only mRFP (red) was detected. After ARCSP or chloroquine (CQ) treatment, the number of autophagosomes was greater than the number of autophagolysosomes. In addition, rapamycin (Rapa)-induced autophagic flux was blocked by ARCSP. These results strongly indicate that ARCSP blocks autophagic flux in HeLa and CaSki cells and that ARCSP-induced autophagic flux inhibition is independent of the mechanisms of ARCSP-initiated autophagy (Fig. 4d).

### ARCSP-mediated cytotoxic death due to excessive accumulation of autophagosomes

**Fig. 5:**
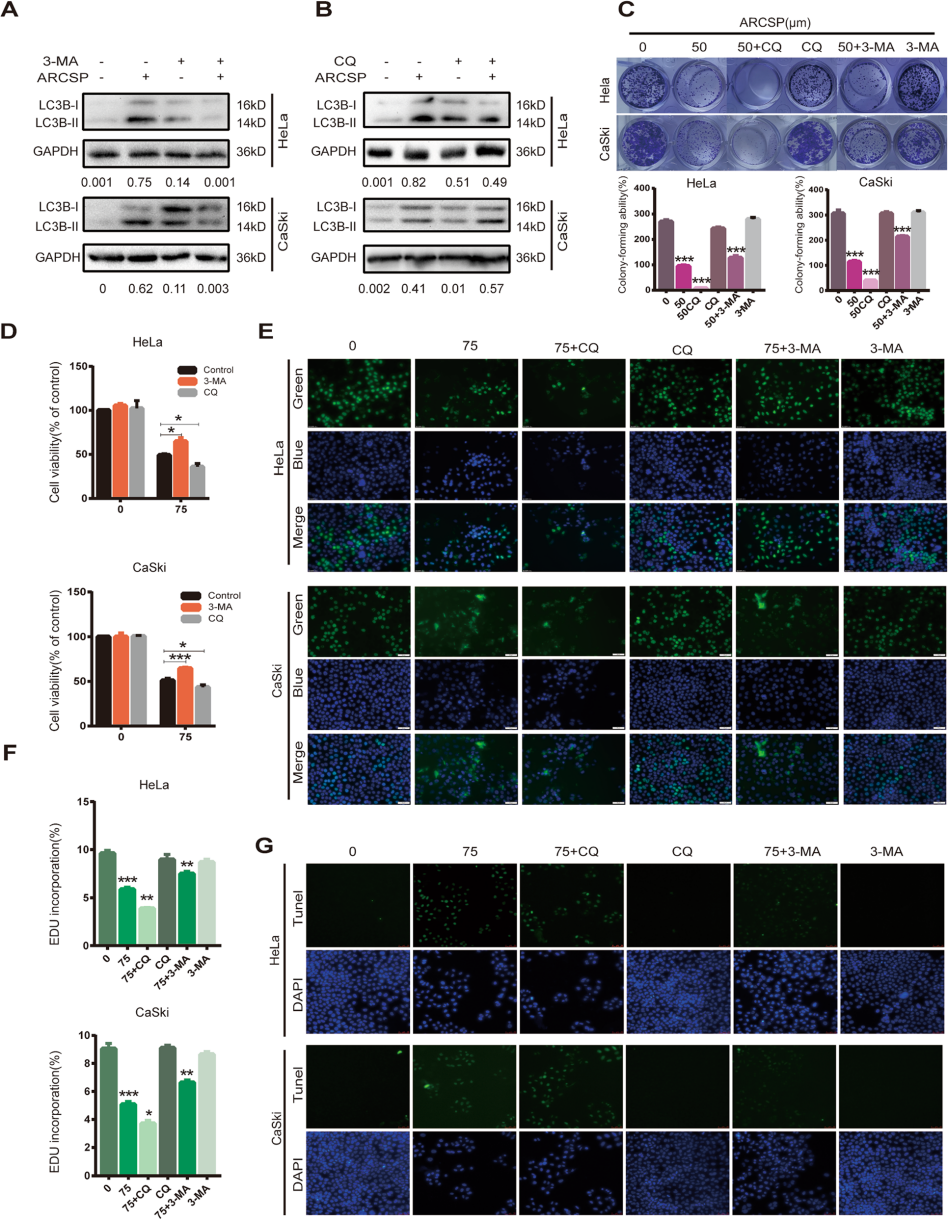
ARCSP-induced cytotoxic death may be associated with the accumulation of autophagosomes. **a**–**b** Cells were treated with ARCSP (75 μM) and 3-MA (20 μM) or CQ (20 μM) for 48 h, and the level of LC3B- II/ I was detected by Western blotting. **c** Cells were treated with ARCSP (50 μM) and 3-MA (20 μM) or CQ (20 μM) for 14 days, and the ability of the cells to proliferate was examined by the cell colony formation assay. The histograms show the quantified results of the colony formation assay, which were calculated using ImageJ software. **d**-**g** Cells were treated with ARCSP (75 μM) and 3-MA (20 μM) or CQ (20 μM) for 48 h. **d** Cell viability was analyzed by the CCK8 assay. **e** The cell proliferation rate was measured by the EdU assay, and the cells were photographed under a fluorescence microscope. Scale bar = 50 μm. **f** The histograms show the quantified results of the EdU assay, which were calculated using ImageJ software. **g** The apoptosis index was determined by the TUNEL assay, and the cells were photographed under a fluorescence microscope. Scale bar = 50 μm. The data are expressed as the mean ± SD; ^*^P < 0.05, ^**^P < 0.01, ^***^P < 0.001. ns, not significant

To confirm the role of ARCSP in blocking autophagic flux in HeLa and CaSki cells, we used an autophagosome formation inhibitor (early autophagy inhibitor, 3-MA) and an autophagosome-lysosome fusion inhibitor (late autophagy inhibitor, CQ). We found that 3-MA significantly reversed the change in the LC3B- II/ I ratio induced by treatment with ARCSP in HeLa and CaSki cells, whereas CQ did not have significant effects on the ratio of LC3B- II/ I (Fig. 5a, b). In addition, the combination of ARCSP and 3-MA treatment reversed the inhibitory effects of ARCSP on the proliferation and colony-forming ability of the cells.

However, the combination of ARCSP and CQ had enhanced the inhibitory effects of ARCSP on the proliferation and colony-forming ability of the cells (Fig. 5c, d, e, f). Consistently, the TUNEL assay showed that the combination of ARCSP and CQ had a significant apoptotic effect on cells (Fig. 5g). These data suggest that ARCSP can block the fusion of autophagosomes and lysosomes, causing the excessive accumulation of autophagosomes and thus enhancing cytotoxic death of HeLa and CaSki cells.

### ARCSP treatment inhibits lysosomal activity

**Fig. 6:**
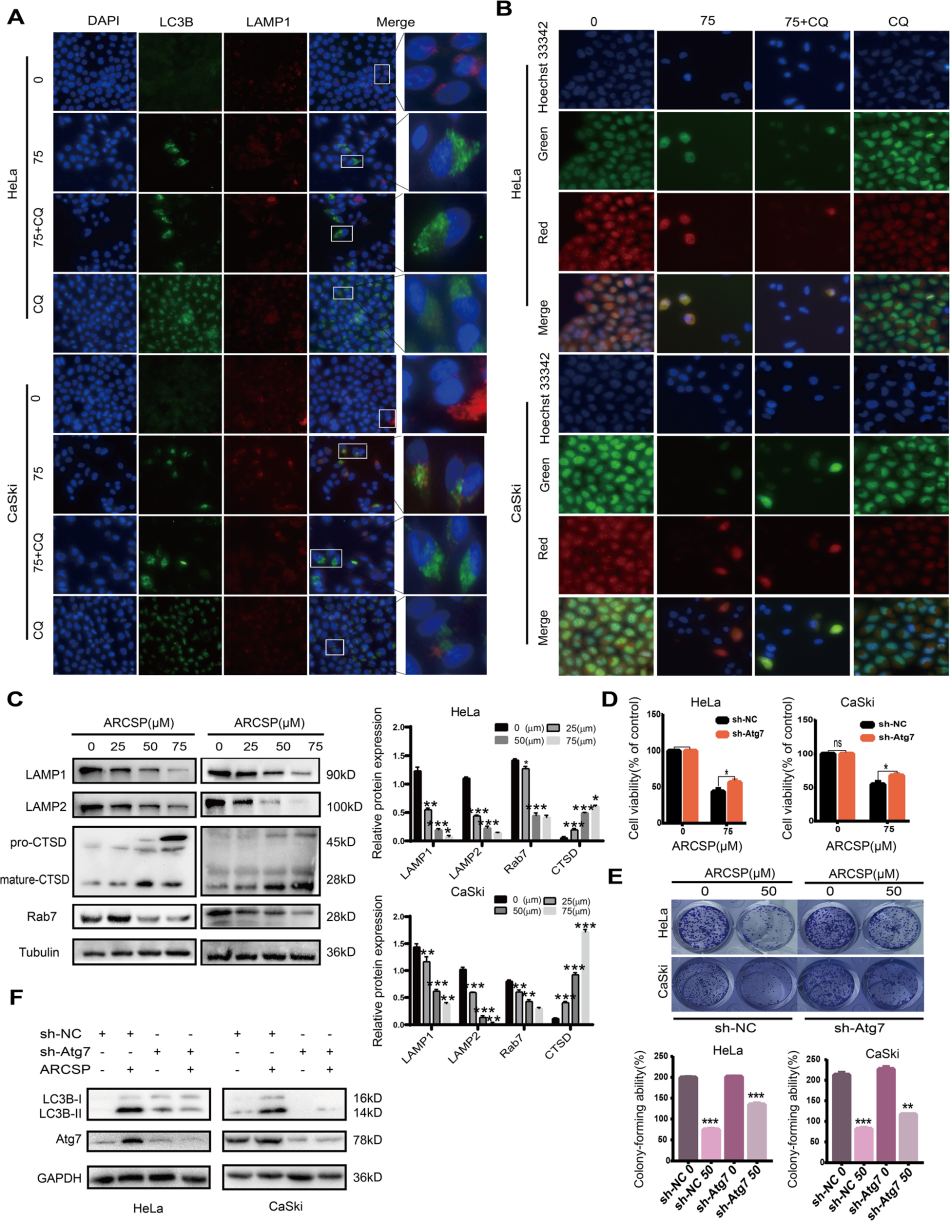
ARCSP may have a cytotoxic effect by affecting the function of lysosomes. **a** Cells were treated with ARCSP (75 μM) for 48 h, and the colocalization of LC3B (488 green) and LAMP1 (594 red) was assessed. DAPI (blue) was used to stain the nuclei, and the cells were photographed under a fluorescence microscope. Scale bar = 25 μm. **b** Cells were treated with ARCSP (75 μM) or CQ (20 μM) for 48 h, stained with AO for 10 min, Hoechst 33342 (blue) was used to stain the nuclei, and photographed under a fluorescence microscope. Scale bar = 25 μm. **c** Cells were treated with ARCSP (0–75 μM) for 48 h, and the levels of Rab7, LAMP1, LAMP2 and CTSD were detected by Western blotting. **d** Cells were transfected with sh-Atg7 and treated with ARCSP (75 μM) for 48 h, and cell viability was analyzed by the CCK8 assay. **e** Cells were transfected with sh-Atg7 and treated with ARCSP (50 μM) for 14 days, and the cell colony formation assay was used to evaluate cell proliferation. The histograms show the quantified results of the colony formation assay, which were calculated using ImageJ software. **f** Cells were treated with ARCSP (75 μM) for 48 h, and the expression of LC3B-I and LC3B-II was detected by Western blotting. The data are expressed as the mean ± SD; ^*^P < 0.05, ^**^P < 0.01, ^***^P < 0.001. ns, not significant

Next, we explored whether the autophagosome and lysosome fusion process is impaired. We investigated whether there endogenous LC3B puncta were colocalized with LAMP1 (a lysosome marker) in ARCSP-treated HeLa and CaSki cells. ARCSP treatment resulted in fewer LAMP1 puncta and decreased colocalization of LAMP1 puncta with LC3B puncta (Fig. 6a), indicating that the fusion of autophagosomes and lysosomes was blocked.

To test whether ARCSP adversely affects lysosomal function, we examined the pH of the lysosomes using acridine orange (AO) and Lyso-Tracker Red staining. These dyes are fluorescent dyes that accumulate in acidic spaces, such as lysosomes, and emit red light. Red fluorescence was decreased in ARCSP-treated cells compared to untreated control cells (Fig. 6b, Fig. S4A). These results indicate that ARCSP-induced inhibition of autophagy is dependent on changes in lysosomal pH.

Furthermore, we examined the expression of Rab7, LAMP1 and LAMP2. Immunoblotting showed that the expression of these proteins were decreased by ARCSP in a dose-dependent manner (Fig. 6c), indicating that ARCSP disrupted lysosomal membrane function to block autophagic flux. Cathepsin D (CTSD) is a lysosomal cathepsin that is involved in lysosomal nonspecific protein degradation. As expected, after ARCSP treatment, CTSD expression was downregulated (Fig. 6c), indicating that ARCSP inhibited lysosomal cathepsin activity, resulting in the accumulation of autophagosomes. In addition, ARCSP treatment resulted in an accumulation of CTSD points (Fig. S4B).

To further investigate the relationship between autophagic flux blockade and ARCSP-induced cell death, we constructed Atg7 knockout cells using shRNA technology and then treated them with ARCSP. Cell viability was measured using the CCK8 assay and the colony formation assay. The data indicated that Atg7 knockout reversed the inhibitory effect of ARCSP on cell proliferation (Fig. 6d, e) and attenuated the expression level of LC3B-II (Fig. 6f), indicating that ARCSP-induced cell death was associated with an increase in the number of autophagosomes. Overall, these data suggest that ARCSP-induced autophagic flux inhibition contributes to cell death of HeLa and CaSki cells.

### ARCSP-induced autophagy exerts antitumor effects in a HeLa cell xenograft model

**Fig. 7:**
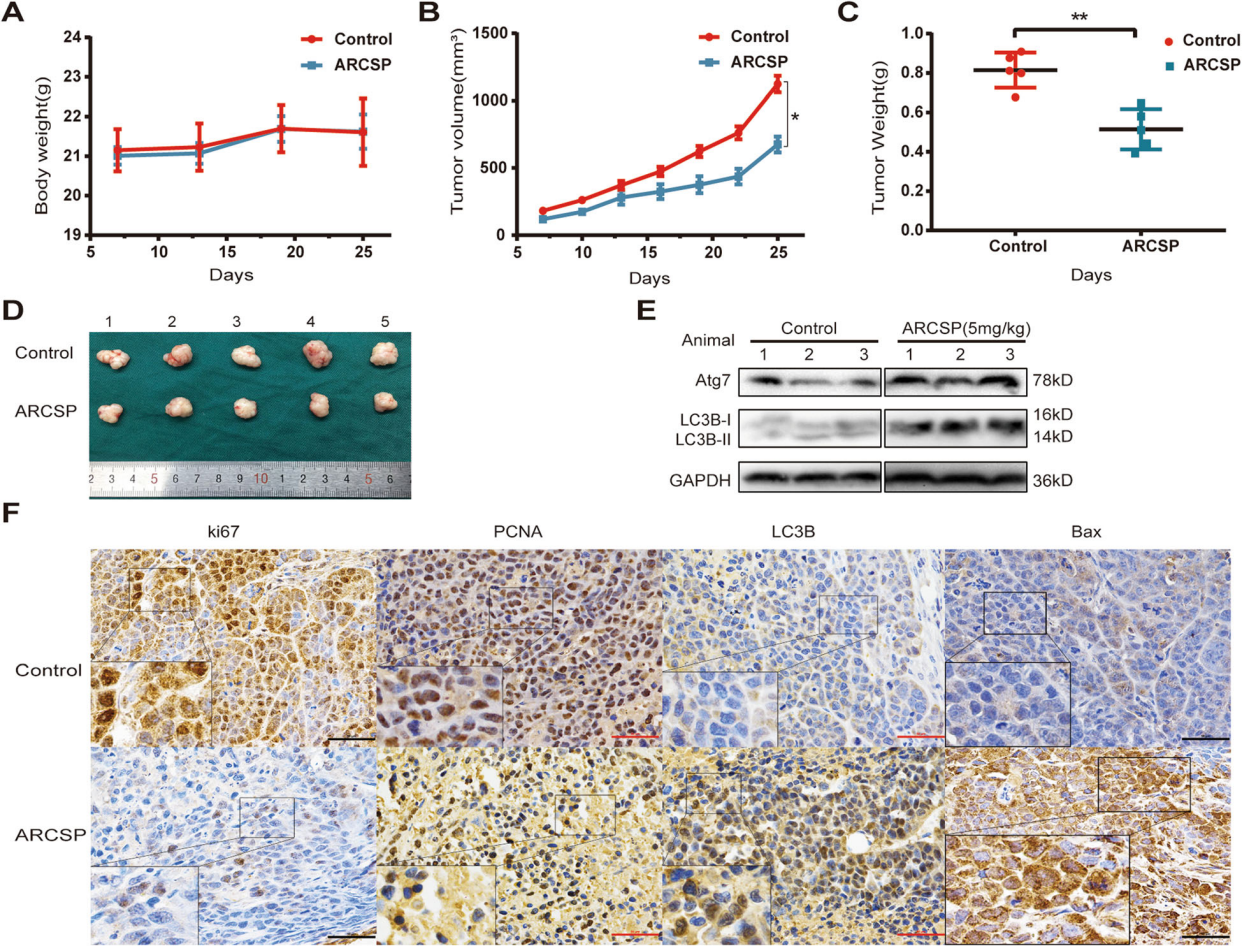
ARCSP-induced autophagy has antitumor effects in vivo*.* **a**-**b** HeLa xenograft tumors in the control group and ARCSP-treated group were measured once a day for 25 days. **a** Body weight change. **b** Tumor volume change. **c** The final tumor weights were measured. **d** An image of a representative tumor is shown. **e** The levels of LC3B-II/I and Atg7 were detected by Western blotting. **f** Immunohistochemical staining with LC3B, Bax, Ki67 and PCNA antibodies in tumor tissue (shown as brown staining). Scale bar = 50 μm. The data are expressed as the mean ± SD; ^*^P < 0.05, ^**^P < 0.01, ^***^P < 0.001. ns, not significant

HeLa cells are more sensitive than other cells to ARCSP in vitro. Therefore, a xenograft model established by using HeLa cells was used to investigate the antitumor activity of ARCSP in vivo. On the 7th day, nude mice were randomly divided into two groups: the control group (intraperitoneally injected with saline) and the treatment group (intraperitoneally injected with ARCSP, 10 mg/kg). Tumors grew more slowly in the ARCSP treatment group than in the control group (Fig. 7a, b). After 18 days of treatment, the nude mice were sacrificed. The final tumor weight of the ARCSP-treated group was significantly different from that of the control group (Fig. 7c). The tumor size of the control group gradually increased with time (Fig. 7d). No change in body weight was observed after ARCSP treatment (Fig. 7a), indicating that ARCSP had almost no toxic effects in vivo.

A portion of the tumor tissue was snap frozen for Western blotting, and another portion was fixed for immunohistochemical staining. Consistent with our in vitro findings, the results showed that the expression of LC3B-II/I and Atg7 was increased in the treatment group compared with the control group (Fig. 7e), indicating that autophagosome accumulation was also induced in vivo. Immunohistochemical analysis revealed that compared with tissues from the control group, tissues from the ARCSP-treated group showed an increase in LC3B and Bax expression and a decrease in Ki67 and PCNA expression (Fig. 7f), further confirming the increase in the number of autophagosomes and the inhibition of proliferation.

## Discussion

Recently, autophagy has emerged as a promising target for cancer treatment, and induction of autophagy-associated cytotoxic death through blockade of autophagic flux has been increasingly recognized as a novel cancer therapeutic strategy [19–21]. In the present study, we indicated that ARCSP has an effective antitumor effect on cervical cancer cells. ARCSP inhibited the growth of cells by inhibiting cell proliferation and inducing apoptosis. Interestingly, we found that ARCSP was a potent autophagy regulator in cells. ARCSP initiates autophagy by activating AMPK/mTOR signaling and impairs autophagic flux through inhibiting lysosomal activity, leading to the accumulation of nonfused autophagolysosomes in cells. Moreover, our findings indicated that the accumulation of nonfused autophagolysosomes by ARCSP enhanced cytotoxic death. We demonstrated for the first time that ARCSP can induce cytotoxic death through blocking autophagic flux and inducing the accumulation of nonfused autophagosomes.

Autophagy is a highly conserved catabolic process though which unnecessary substances or dysfunctional cellular components are captured and degraded in autophagolysosomes for recycling [22]. Autophagy has two functions in cancer therapy [23–25]. On the one hand, autophagy may induce cell death [26, 27]; on the other hand, autophagy may protect cells against external factors, such as drug resistance [28, 29]. In the present study, we demonstrated that ARCSP can induce autophagosome synthesis. However, due to the lysosome dysfunction induced by ARCSP, autophagosomes and lysosomes cannot be normally fused and degraded. Blockade of the early initiation of autophagy reversed ARCSP-induced cervical cancer proliferation inhibition, while inhibiting the fusion of autophagosomes and lysosomes aggravated ARCSP-induced apoptosis.

Our results indicate that ARCSP inhibits the proliferation of cervical cancer cells and induces apoptosis in vitro and in vivo (Figs. 1, 2 and 7). Moreover, we observed an increase in the number of autophagosomes by electron microscopy (Fig. 3b). ARCSP induced high expression of p-AMPK, p-raptor, LC3B-II, atg7, and p62 and low expression of p-mTOR and p-p70s6k in cervical cancer cells (Figs. 3 and 4a). However, the p62 protein is an autophagy substrate that binds to LC3B-II in autophagosomes and is degraded after the formation of autophagolysosomes [30]. The observed increase in P62 protein expression indicated that autophagic flux was blocked (Fig. 4c, d). In addition, our further experiments showed that blocking autophagy flux strongly inhibited cell proliferation and enhanced cell death (Fig. 5). Given that ARCSP treatment leads to the accumulation of autophagosomes (increased levels of LC3B-II), this may represent either an increase in the number of autophagosomes or blockade of the formation of autophagolysosomes through the fusion of autophagosomes and lysosomes [31]. We assumed that ARCSP can increase the number of autophagosomes in cells by increasing autophagosome formation and blocking autophagolysosomal degradation. Our results confirmed that ARCSP induced an increase in lysosomal pH (Fig. 6b, Fig. S4A) and inactivation of CTSD (Fig. 6c), which affected lysosomal function. We demonstrated that ARCSP blocked autophagic flux, leading to the accumulation of nonfusion autophagosomes and enhancement of cell death. Therefore, we identified ARCSP as a novel lethal nonfused autophagosome inducer with low cytotoxicity to normal cells (Fig. 1c).

As a key cellular energy sensor, AMPK plays an important role in activating autophagy by inhibiting the activity of mTOR [32]. In addition to initiating autophagy, AMPK also plays an important role in the late stages of autophagy. On the one hand, AMPK can increase the ATP level in cells to promote autophagic degradation and maintain homeostasis; on the other hand, when intracellular nutrients are consumed excessively, negative feedback by AMPK inhibits autophagy because autophagy requires a sufficient amount of ATP to complete this complex membrane-dependent process [33–36]. Furthermore, the application of the AMPK inhibitor CC combined with ARCSP treatment significantly reversed the expression of LC3B-II (Fig. 3e), indicating that activation of AMPK caused autophagosome formation after ARCSP treatment.

Autophagy is a basic catabolic process during which damaged cellular tissues or dysfunctional proteins are engulfed by autophagosomes, which then fuse with lysosomes to mediate degradation to maintain cell homeostasis [37]. Lysosomes are considered the degradation centers of most eukaryotic cells [38, 39]. Lysosomal acidification, lysosomal transport, fusion of lysosomes with late endosomes or autophagosomes, and maturation of lysosomal proteases are essential for the maintenance of normal lysosomal physiology. Therefore, inhibition of lysosomal and autophagolysosomal functions severely impairs autophagic flux [40]. Our results indicated that after treatment with ARCSP, lysosomes and autophagosomes could not fuse properly, resulting in the accumulation of nonfused autophagosomes (Fig. 4d). In addition, ARCSP treatment increased the lysosomal pH (Fig. 6b, Fig. S4A) and downregulated the expression of LAMP1, LAMP2, Rab7, and CTSD (Fig. 6c), indicating that it severely affected the lysosomal function. Rab7, LAMP1 and LAMP2 are key membrane proteins on lysosomes and play important roles in maintaining the normal physiological function of lysosomes. CTSD is a lysosomal cathepsin that is involved in nonspecific protein degradation in lysosomes. Inhibition of CTSD function leads to massive accumulation of nonfused autophagosomes [41]. It has been confirmed that blockade of autophagic flux can cause the accumulation of dysfunctional proteins or damaged organelles, which can be fatal for cancer cells [40]. Notably, the effects of ARCSP were similar to those of CQ, which inhibits autophagosome-lysosome fusion. This is consistent with some recent studies showing that disruption of the late stage of autophagy leads to excessive accumulation of nonfused autophagosomes and has the potential to turn autophagy into a destructive process, resulting in fatal toxic effects on cells [30, 31].

Autophagy inhibitors have been shown to be useful adjuvants for many antitumor therapies in vivo [42]. The combination of CQ or its derivatives and chemotherapeutics has been used in phase 1 and phase 2 clinical trials for a variety of tumors [43, 44]. Therefore, we chose cisplatin (CDDP), a widely used chemotherapy drug for cervical cancer. The results of the CCK8 assay showed that ARCSP enhanced the lethality of low doses of CDDP (2.5-10 μM) and that lethality increased in an ARCSP dose-dependent manner. The survival rates of HeLa and CaSki cells following treatment with low-dose cisplatin (2.5 μM) for 48 h were 69.71 ± 0.99% and 73.30 ± 2.72%, respectively. After treatment with low doses of ARCSP (50 μM) for 24 h, the survival rates of HeLa and CaSki cells were only 46.15 ± 2.33% and 58.28 ± 2.40%, respectively (Fig. S5A-D). Therefore, the combination of ARCSP and chemotherapeutic drugs needs to be further studied.

## Conclusion

In summary, we discovered a novel mechanism of ARCSP-induced cervical cancer cell death and demonstrated that ARCSP-mediated inhibition of autophagic flux is key to the toxic death of cervical cancer cells. Mechanistic studies showed that ARCSP increased the number of nonfused autophagosomes in two ways: on the one hand, ARCSP induced apoptosis through mitochondrial dysfunction and initiated autophagy by activating the AMPK/mTOR signaling pathway; on the other hand, ARCSP impaired endosome maturation and lysosomal cathepsin activity to block autophagic flux, resulting in the accumulation of nonfused autophagosomes and thus leading to cytotoxic death. Identifying natural compounds that regulate autophagy may be an effective method for identifying novel autophagy inhibitors and lead compounds for cancer treatment [37, 45, 46]. Our results indicate that ARCSP can be used as an autophagy-targeted drug and low-dose chemotherapy adjuvant for cervical cancer with great research and application potential.

## Supplementary information

**Additional file 1: Figure S1.** ARCSP was detected by HPLC and MS. (A) After synthesis, ARCSP was subjected to HPLC. Pure ARCSP was observed at a retention time of 11.962 min; the proportion of this peptide was 95.3198%. (B) The evidence of identity based on mass spectral characterization.

**Additional file 2: Figure S2.** ARCSP induced cell apoptosis and increased intracellular ROS levels in cells. (A) Cells were treated with ARCSP (0-75 μM), the ratio of cell apoptosis was determined by flow cytometry. (B) Cells were cotreated with ARCSP (75 μM) and NAC (5 μM) for 48 h and incubated with 1 mL of serum-free medium containing DCFH-DA for 30 min. The amount of ROS produced was measured under a fluorescence microscope. Scale bar = 25 μm. The histograms show the quantified results of ROS localization, which were calculated using ImageJ software. (C) Cells were cotreated with ARCSP (75 μM) and NAC (5 μM) for 48 h and incubated with 1 mL of serum-free medium containing DCFH-DA for 30 min. ROS production was determined by flow cytometry. (D) Cells were cotreated with ARCSP (75 μM) and NAC (5 μM) for 14 days, and clone-forming ability was determined by the cell colony formation assay. (E) After HeLa and CaSki cells were treated with ARCSP (0-75 μM) for 48 h, we detected the expression of Raptor and p-Raptor by Western blotting. The data are expressed as the mean ± SD; ^*^P < 0.05, ^**^P < 0.01, ^***^P < 0.001. ns, not significant.

**Additional file 3: Figure S3.** Autophagy flux is blocked by ARCSP. Cells were treated with ARCSP (75 μM) for 48 h and subjected to colocalization analysis of LC3B (488, green) and p62 (594, red). DAPI (blue) was used to stain the nuclei, and the cells were photographed under a fluorescence microscope. Scale bar = 25 μm.

**Additional file 4: Figure S4.** ARCSP treatment inhibits lysosomal activity. (A) Cells were treated with ARCSP (75 μM) or CQ (20 μM) for 48 h, stained with Lyso Tracker-Red for 40 min, Hoechst 33342 (blue) was used to stain the nuclei, and photographed under a fluorescence microscope. Scale bar = 50 μm. (B) Cells were treated with ARCSP (75 μM) for 48 h, immunolabeling with CTSD (488 green) antibodies. DAPI (blue) was used to stain the nuclei, and the cells were photographed under a fluorescence microscope. Scale bar = 25 μm. (C) After HeLa and CaSki cells were treated with ARCSP (0-75 μM) for 48 h, we detected the expression of Galectin-3 by Western blotting.

**Additional file 5: Figure S5.** The combined therapy of ARCSP and cisplatin in HeLa and CaSki cells. (A) The HeLa and CaSki cells were treated with CDDP (0-15 μM) for 48 h, and cell viability was measured by CCK8 assay. (B) The HeLa and CaSki cells were co-treated with CDDP (2.5 μM, 5 μM, 10 μM) or ARCSP (0-100 μM) for 24 h, and cell viability was measured by CCK8 assay. The data are expressed as the mean ± SD; ^*^P < 0.05, ^**^P < 0.01, ^***^P < 0.001. ns, not significant.

## Data Availability

The data supporting the findings of this study are included in this paper and its additional files.

## Abbreviations

ARCSP: Autophagy-related cancer suppressing peptide
FITC: Fluorescein isothiocyanate
ARCSP-FITC: Autophagy-related cancer suppressing peptide labeled with fluorescein isothiocyanate
CCK8: Cell counting kit-8
LDH: Cytotoxicity lactate dehydrogenase release
EDU: 5-ethynyl-20-deoxyuridine
TUNEL: TdT-mediated dUTP Nick-End Labeling
AO: Acridine orange
QRT-PCR: Reverse transcription-polymerase chain reaction and real-time PCR
AMPK: Adenosine 5′-monophosphate activated protein kinase
mTOR: Mammalian target of rapamycin
PI: Propidium
DAPI: 4′, 6′-diamidino-2-phenylindole
LC3B: Microtubule associated protein 1 light chain 3 β
ATG7: Autophagy-related 7
ROS: Reactive oxygen species
ATP: Adenosine triphosphate
HPLC: High performance liquid chromatography
MS: Mass spectrometry
CQ: Chloroquine
NAC: *N*-acetyl-L-cysteine
3-MA: 3-Methyladenine
Rapa: Rapamycin
CC: Compound C
DCFH-DA: Oxidation-sensitive probe dichlorodihydro-fluorescein diacetate
LAMP1: Recombinant lysosomal associated membrane protein 1
LAMP2: Recombinant lysosomal associated membrane protein 2
CTSD: Cathepsin D
PCNA: Proliferating cell nuclear antigen
APOC1: Apolipoprotein C
