# Supplementary figures and images for: Lysosomal dysfunction and autophagy blockade contribute to autophagy-related cancer suppressing peptide-induced cytotoxic death of cervical cancer cells through the AMPK/mTOR pathway
Source: J Exp Clin Cancer Res. 2020 Sep 22;39:197. doi: 10.1186/s13046-020-01701-z (PMC7510096; doi:10.1186/s13046-020-01701-z)

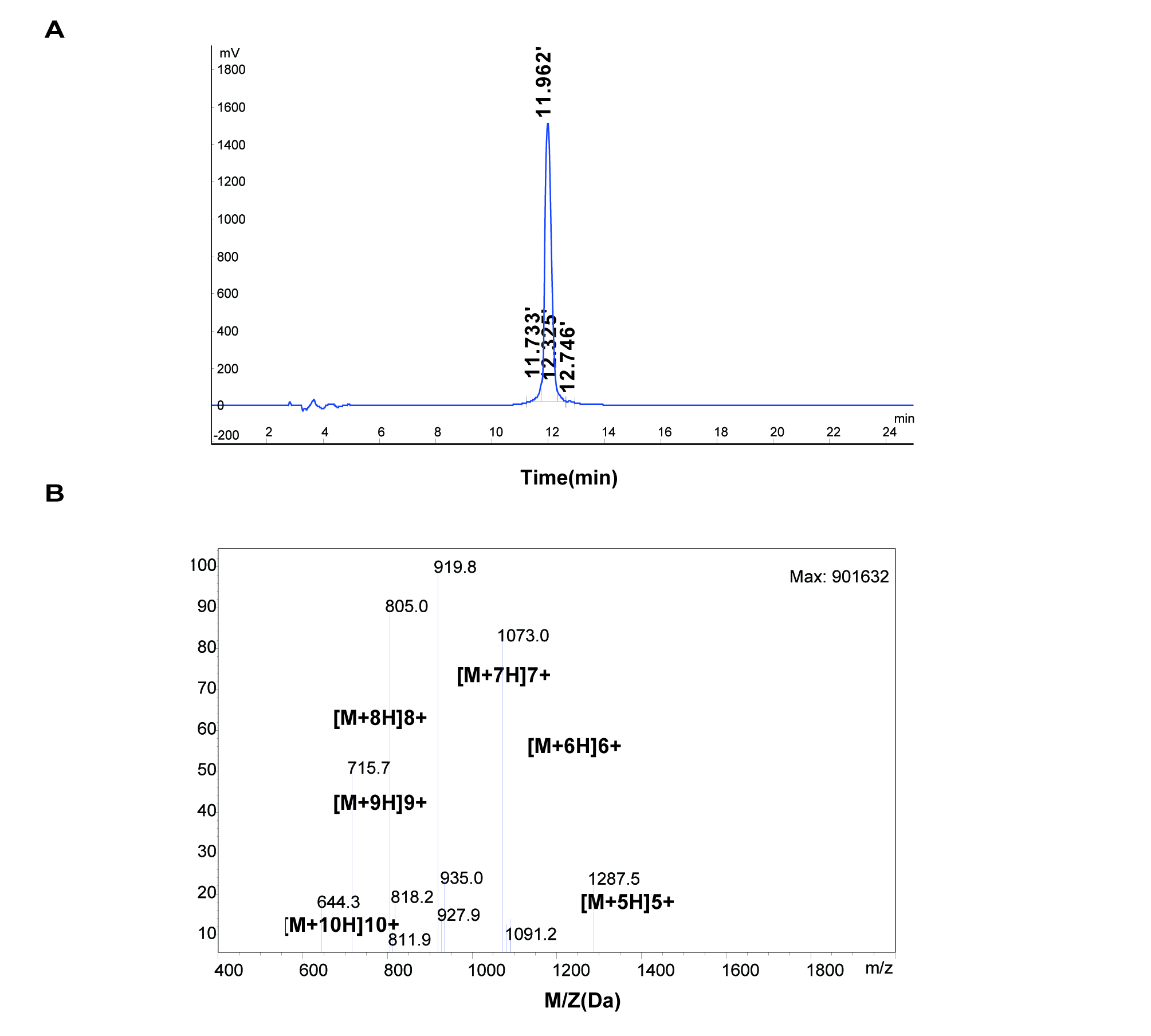

Supplement: Supplementary file 1 — Additional file 1: Figure S1. ARCSP was detected by HPLC and MS. (A) After synthesis, ARCSP was subjected to HPLC. Pure ARCSP was observed at a retention time of 11.962 min; the proportion of this peptide was 95.3198%. (B) The evidence of identity based on mass spectral characterization. [file 13046_2020_1701_MOESM1_ESM.tif]

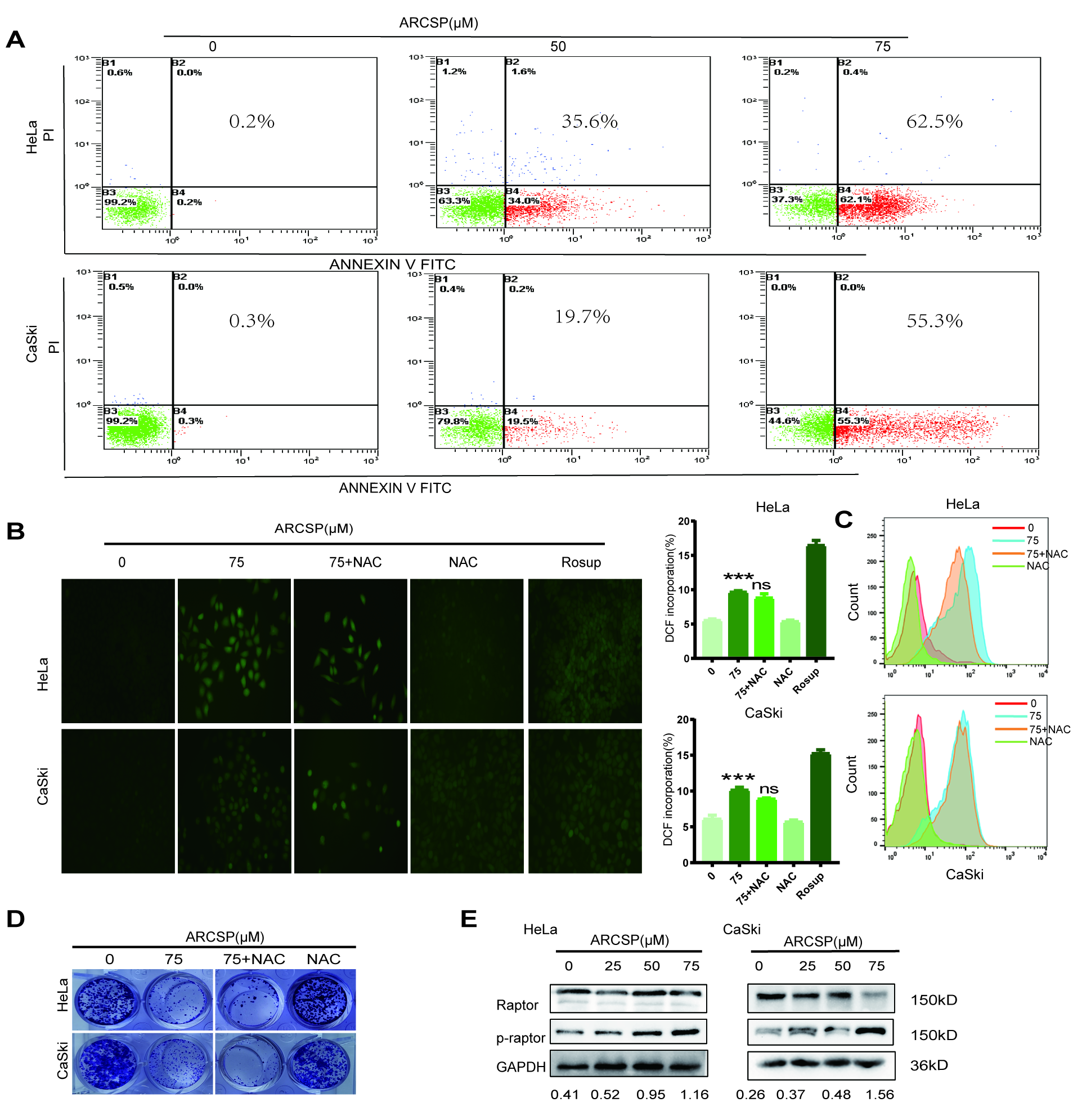

Supplement: Supplementary file 2 — Additional file 2: Figure S2. ARCSP induced cell apoptosis and increased intracellular ROS levels in cells. (A) Cells were treated with ARCSP (0-75 μM), the ratio of cell apoptosis was determined by flow cytometry. (B) Cells were cotreated with ARCSP (75 μM) and NAC (5 μM) for 48 h and incubated with 1 mL of serum-free medium containing DCFH-DA for 30 min. The amount of ROS produced was measured under a fluorescence microscope. Scale bar = 25 μm. The histograms show the quantified results of ROS localization, which were calculated using ImageJ software. (C) Cells were cotreated with ARCSP (75 μM) and NAC (5 μM) for 48 h and incubated with 1 mL of serum-free medium containing DCFH-DA for 30 min. ROS production was determined by flow cytometry. (D) Cells were cotreated with ARCSP (75 μM) and NAC (5 μM) for 14 days, and clone-forming ability was determined by the cell colony formation assay. (E) After HeLa and CaSki cells were treated with ARCSP (0-75 μM) for 48 h, we detected the expression of Raptor and p-Raptor by Western blotting. The data are expressed as the mean ± SD; *P < 0.05, **P < 0.01, ***P < 0.001. ns, not significant. [file 13046_2020_1701_MOESM2_ESM.tif]

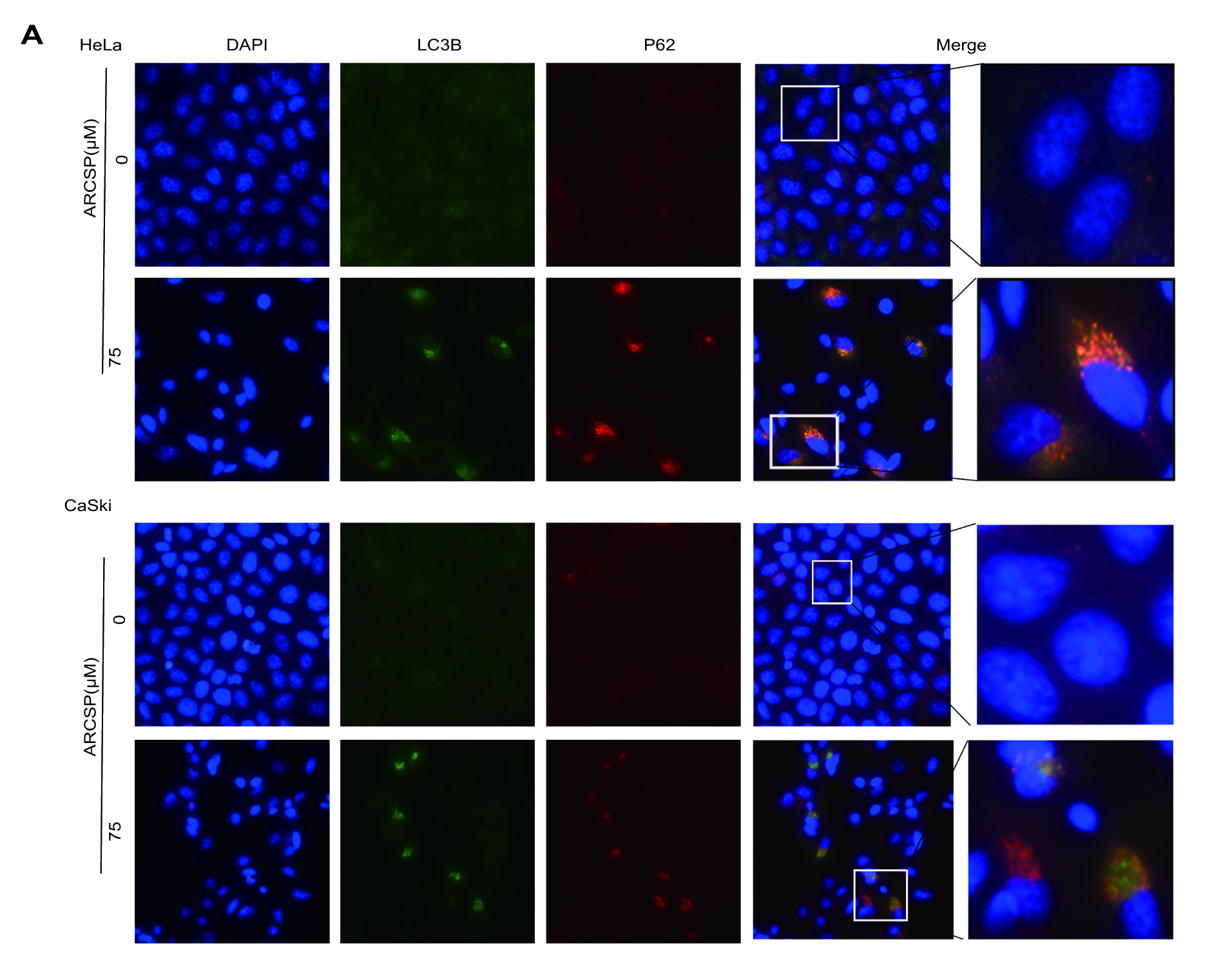

Supplement: Supplementary file 3 — Additional file 3: Figure S3. Autophagy flux is blocked by ARCSP. Cells were treated with ARCSP (75 μM) for 48 h and subjected to colocalization analysis of LC3B (488, green) and p62 (594, red). DAPI (blue) was used to stain the nuclei, and the cells were photographed under a fluorescence microscope. Scale bar = 25 μm. [file 13046_2020_1701_MOESM3_ESM.tif]

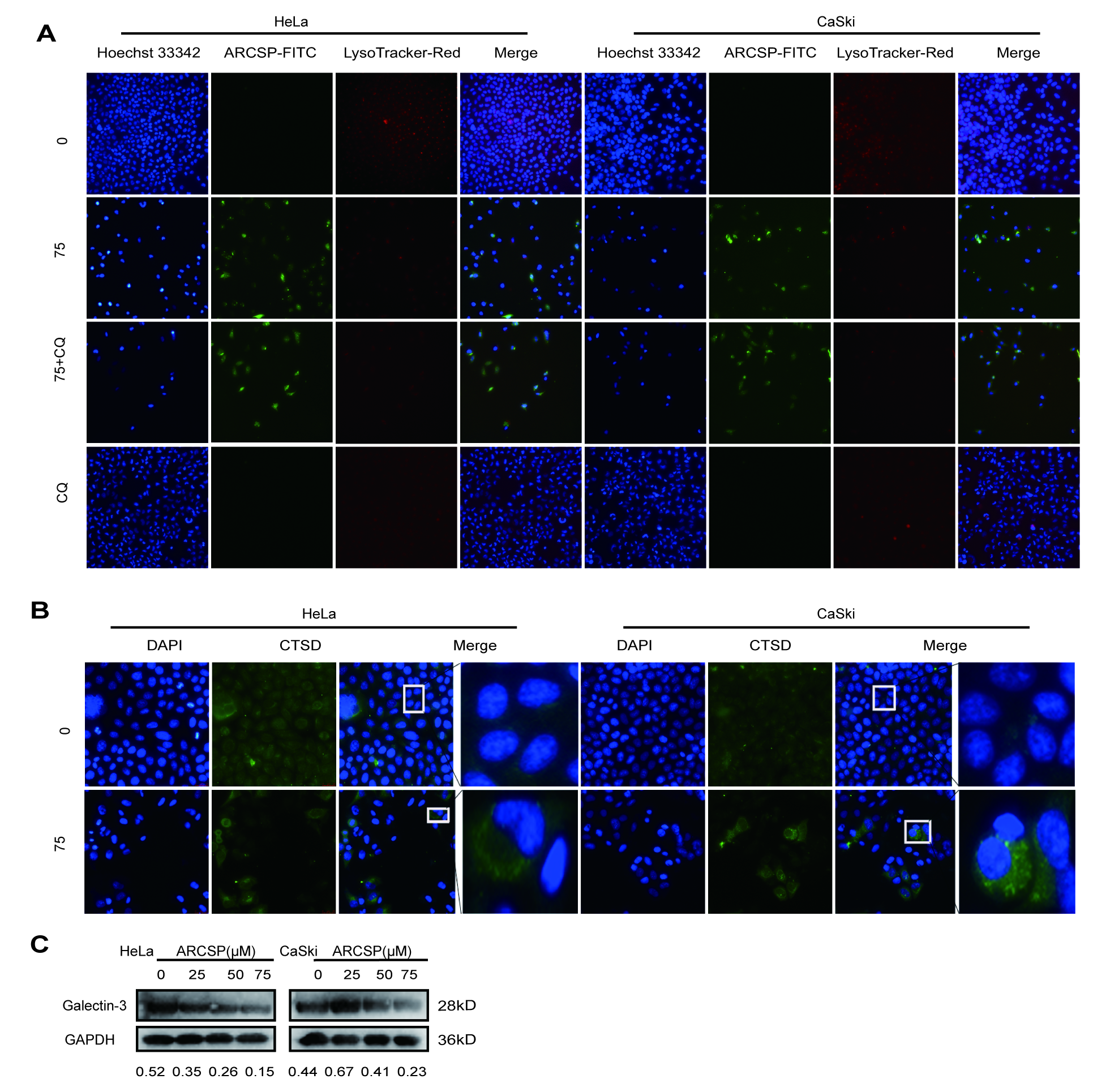

Supplement: Supplementary file 4 — Additional file 4: Figure S4. ARCSP treatment inhibits lysosomal activity. (A) Cells were treated with ARCSP (75 μM) or CQ (20 μM) for 48 h, stained with Lyso Tracker-Red for 40 min, Hoechst 33342 (blue) was used to stain the nuclei, and photographed under a fluorescence microscope. Scale bar = 50 μm. (B) Cells were treated with ARCSP (75 μM) for 48 h, immunolabeling with CTSD (488 green) antibodies. DAPI (blue) was used to stain the nuclei, and the cells were photographed under a fluorescence microscope. Scale bar = 25 μm. (C) After HeLa and CaSki cells were treated with ARCSP (0-75 μM) for 48 h, we detected the expression of Galectin-3 by Western blotting. [file 13046_2020_1701_MOESM4_ESM.tif]

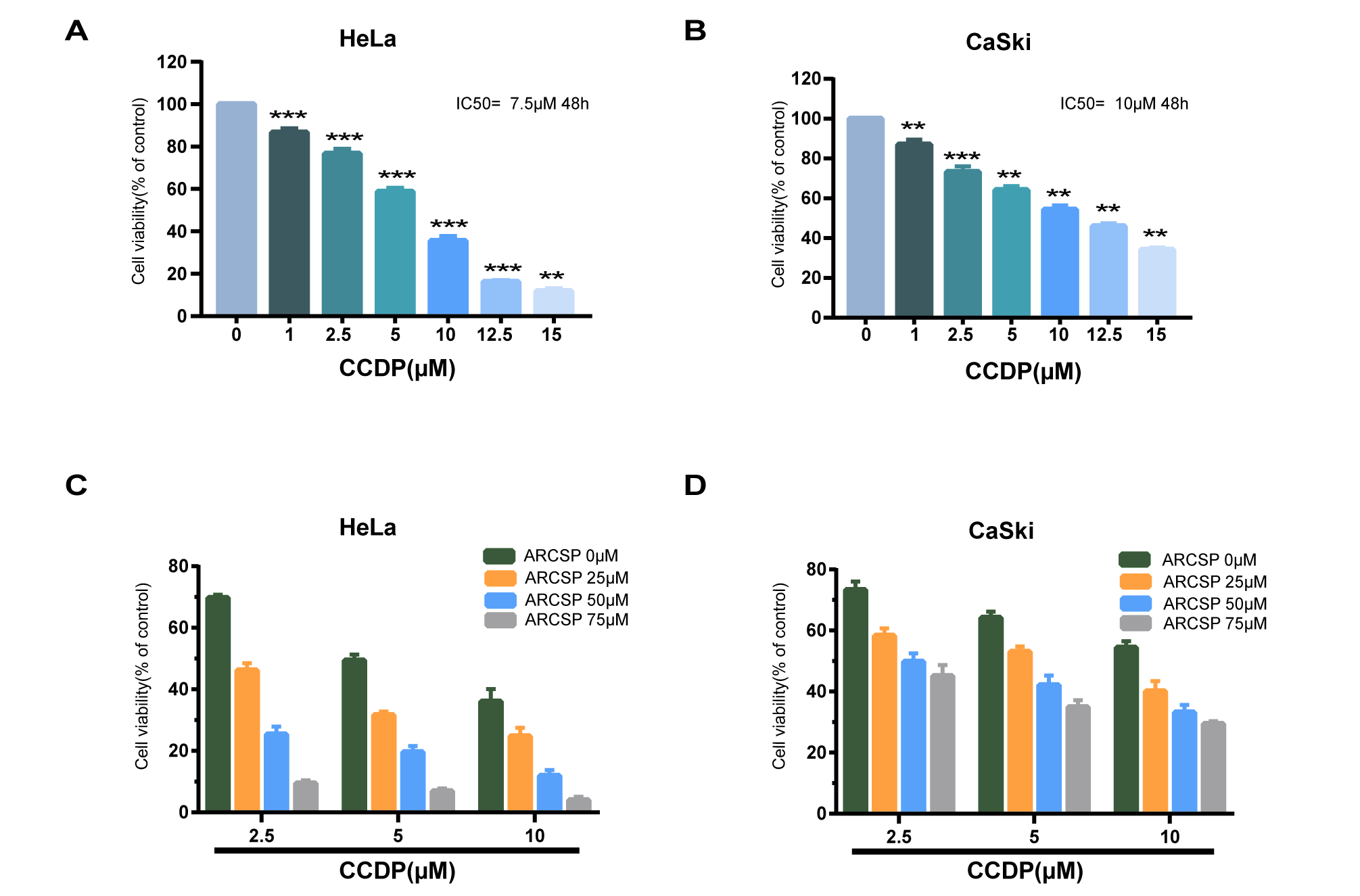

Supplement: Supplementary file 5 — Additional file 5: Figure S5. The combined therapy of ARCSP and cisplatin in HeLa and CaSki cells. (A) The HeLa and CaSki cells were treated with CDDP (0-15 μM) for 48 h, and cell viability was measured by CCK8 assay. (B) The HeLa and CaSki cells were co-treated with CDDP (2.5 μM, 5 μM, 10 μM) or ARCSP (0-100 μM) for 24 h, and cell viability was measured by CCK8 assay. The data are expressed as the mean ± SD; *P < 0.05, **P < 0.01, ***P < 0.001. ns, not significant. [file 13046_2020_1701_MOESM5_ESM.tif]
